# Bilateral low origin of testicular artery: a case report

**DOI:** 10.1590/1677-5449.003017

**Published:** 2017

**Authors:** Satheesha Nayak Badagabettu, Swamy Ravindra Shantakumar, Surekha Devadas Shetty, Gayathri Prabhu

**Affiliations:** 1 Manipal University, Melaka Manipal Medical College, Department of Anatomy, Manipal, Karnataka, India.

**Keywords:** testicular artery, low origin, angiography, variation, artéria testicular, origem baixa, angiografia, variação

## Abstract

The testicular artery is frequently subjected to radiographic imaging techniques such as angiography for diagnosis and treatment of conditions like epididymitis, testicular torsion, tumor, hematoma, and hydrocele and in cases of undescended testis. Radiologists and surgeons should therefore be aware of testicular artery variants. Although there are numerous studies and case reports that mention testicular artery variants, this is probably the first case, reporting a bilateral low origin of the testicular artery and discussing its probable embryological etiology.

## Resumo

A artéria testicular é frequentemente examinada por técnicas de imagem como angiografia, para o diagnóstico e tratamento de condições como epididimite, torsão testicular, tumor, hematoma e hidrocele, e também em casos de criptorquidia. Sendo assim, radiologistas e cirurgiões devem estar cientes de variantes anatômicas da artéria testicular. Embora numerosos estudos e relatos de caso mencionem variantes da artéria testicular, este provavelmente é o primeiro caso a descrever origem bilateral baixa da artéria testicular e a discutir sua provável etiologia embriológica.

## INTRODUCTION

**Figure 1:**
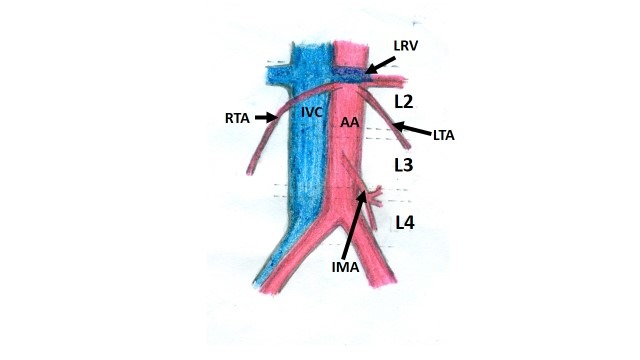
Schematic diagram showing the normal right and left testicular artery (RTA & LTA) originating from the abdominal aorta (AA) at the level of the second lumbar vertebra, just below the renal vessels. IMA, inferior mesenteric artery; IVC, inferior vena cava; RTV, right testicular vein.

The testes are supplied by testicular arteries that arise from the ventral aspect of the abdominal aorta, at the level of the second lumbar vertebra, just below the origin of the renal arteries (Figure 1).^1^ They descend obliquely behind the peritoneum to reach the pelvic cavity.^2^ Variant origins of the testicular artery have been reported by several authors.^1^ Knowledge of such variations is important from a developmental perspective, for research interest, and for avoiding diagnostic complications during radiological examination or during surgical approach in the region.^2^ We report here a rare variation in which bilateral testicular arteries had a low origin.

## CASE REPORT

**Figure 2:**
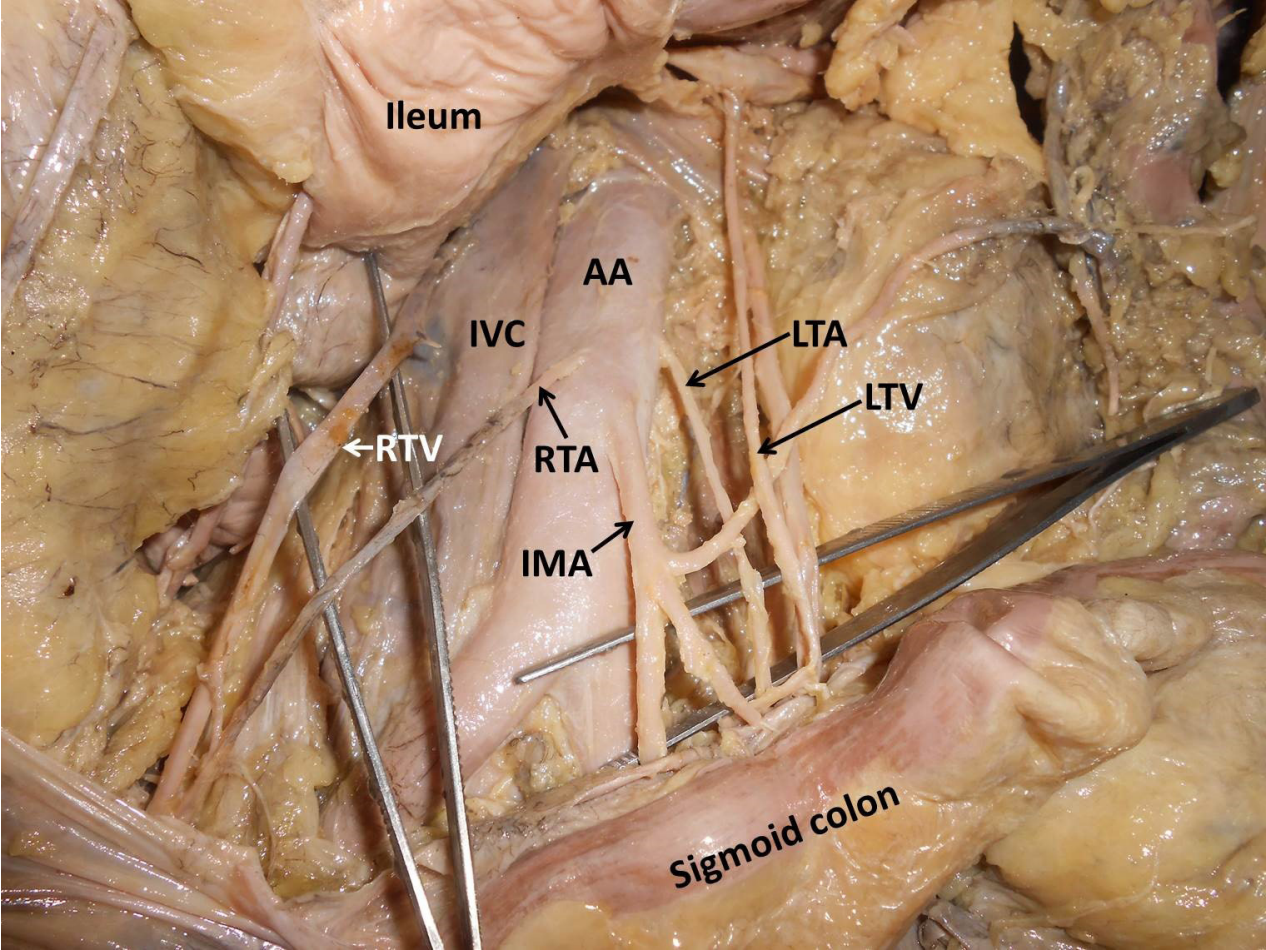
Dissection of posterior abdominal wall showing the major vessels along with the variant right and left testicular arteries (RTA & LTA) originating from the abdominal aorta (AA) just above the origin of the inferior mesenteric artery (IMA). IVC, inferior vena cava; LRV, left renal vessels.

**Figure 3:**
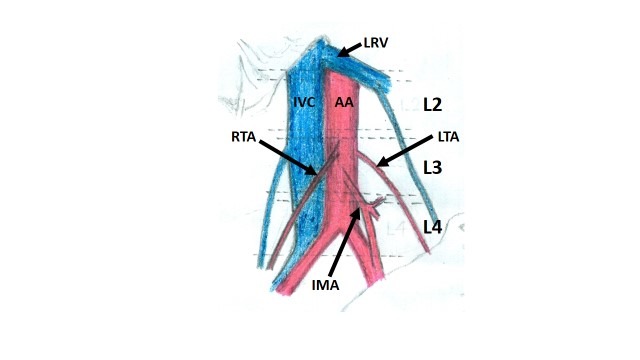
Schematic diagram showing the low origin of right and left testicular arteries (RTA & LTA) from the abdominal aorta (AA) at the level of the third lumbar vertebra, just above the origin of the inferior mesenteric artery (IMA). IMA, inferior mesenteric artery; IVC, inferior vena cava; LRV, left renal vessels.

During routine abdomen dissection class for undergraduate medical students, we came across a variant testicular artery origin in the cadaver of an adult male aged approximately 60 years. Both the testicular arteries had a low origin from the abdominal aorta, at a level corresponding to the third lumbar vertebra. (Figures 2 and 3). The left testicular artery arose from the left lateral edge of the aorta and the right testicular artery arose from the ventral aspect of the abdominal aorta, just above the origin of the inferior mesenteric artery. The left colic branch of the inferior mesenteric artery ran towards the left side, passing in front of the left testicular artery and behind the left testicular vein. All other vessels and structures appeared to be normal.

## DISCUSSION

There are a number of studies and reports in the literature regarding testicular artery variants. Anomalies of the testicular artery in terms of origin, course, and number were observed in 8.8 and 4.7 percent of cases in two studies, most of them with high origin.^2^^,^ 3 The variations reported include double testicular arteries, common origin of both testicular arteries, absence of one testicular artery, a higher origin than normal, or origin from the lumbar artery, renal artery, polar renal arteries, middle or superior suprarenal arteries, common or internal iliac arteries, or superior epigastric artery.^3^ According to Machnicki et al., variations of the testicular artery in both fetuses and adults can be classified into four types by origin: Type A - single testicular artery arising from the aorta, Type B - single testicular artery arising from the renal artery, Type C - two testicular arteries arising from the aorta supplying the same testis, Type D - two testicular arteries, one arising from the aorta and the other arising from the renal artery, supplying the same testis.^4^ Pai et al. found a variant testicular artery in 14.7% of cases in a cadaveric study. A variant testicular artery originated from an inferior polar artery in five cases, from a renal artery in one case, there were three cases of high origin of the artery, and one case of double testicular artery.^5^

In human fetuses, variations in the origin of the gonadal arteries can be classified into four types: Type 1: from the suprarenal artery, Type 2: from the renal artery, Type 3: high origin from the abdominal aorta near the renal artery level, Type 4A: duplication of the testicular arteries, and Type 4B: two right testicular arteries from different vessels.^2^

Numerous case reports of variant testicular artery origins have been published, most of them mentioning high origin. Bergman et al. reported two variant origins of testicular arteries; one from the renal artery, the other from the inferior polar renal artery from the abdominal aorta.^6^ A testicular artery originating 1 cm above the origin of the inferior phrenic artery was reported by Shinohara et al.^7^ A similarly high origin has been reported by other authors as well.^8^^-^ 10 A case in which the left testicular artery was entrapped between two divisions of left renal veins has also been reported.^11^

According to Felix, nine lateral mesonephric arteries of the embryo can be divided into three groups, namely: cranial, middle, and caudal. Any one of these nine lateral mesonephric arteries may eventually become the gonadal artery, although it commonly arises from the caudal group. The existence of a high-positioned gonadal artery has been explained as persistence of a mesonephric artery from the cranial part.^12^ However, in the present case, persistence of the most caudal mesonephric artery may have led to formation of a low testicular artery origin.

Selective injection into the testicular artery for magnification angiography of testicular artery is performed in patients with a variety of different lesions of the scrotal structures, such as epididymitis, testicular torsion, tumor, hematoma, and hydrocele.^13^ Magnification testicular angiography can be used to study the vascular anatomy of the testis for diagnosis and determination of the characteristics, location, and nature of both intra and extra testicular masses.^14^ Diagnostic clinicians and surgeons may come across low origin of testicular artery during angiography for radiological diagnosis and treatment of undescended testis or cryptorchidism.^15^^,^ 16

It has been reported that hemorrhagic complications following retroperitoneal operation are possible in cases of gonadal vascular variations.^17^^,^ 18 The present case of low origin of testicular artery could lead to iatrogenic injuries during lower abdominal surgeries. In our survey of the literature, we did not find any reports of bilateral low origin of testicular arteries. While there are reports on variant origins of testicular arteries, the present case of low origin can be considered an extremely rare variation of the testicular artery.

## CONCLUSION

To the best of our knowledge, this is the first report of bilateral low origin of testicular arteries, which can be clinically and embryologically important.
